# Supplementary material for: Effect of High Fat and Fructo-Oligosaccharide Consumption on Immunoglobulin A in Saliva and Salivary Glands in Rats
Source: Nutrients. 2021 Apr 10;13(4):1252. doi: 10.3390/nu13041252 (PMC8070188; doi:10.3390/nu13041252)
Supplement: Supplementary file 1 [file nutrients-13-01252-s001.zip › Nutrients Supplementary Materials/Nutrients Supplementary Materials.doc]

**Table S1.** The composition of low-fat fiberless diet (LFFL), low-fat fracto-oligosaccharide diet (LFFOS), high-fat fiberless diet (HFFL) and high-fat fracto-oligosaccharide diet (HFFOS) (g/kg)

| **Ingredient** | **LFFF** | **LFFOS** | **HFFF** | **HFFOS** |
| --- | --- | --- | --- | --- |
| Sucrose | 690 | 660 | 530 | 500 |
| Casein | 200 | 200 | 200 | 200 |
| Lard | 60.0 | 60.0 | 220 | 220 |
| Mineral mixture (AIN-76) ^*^ | 35.0 | 35.0 | 35.0 | 35.0 |
| Vitamin mixture (AIN-76) ^†^ | 10.0 | 10.0 | 10.0 | 10.0 |
| DL-Methionine | 3.00 | 3.00 | 3.00 | 3.00 |
| Choline bitartrate | 2.00 | 2.00 | 2.00 | 2.00 |
| FOS |  | 30.0 |  | 30.0 |
| Total | 1000 | 1000 | 1000 | 1000 |

^*^Mineral mixture (AIN-76) (g/kg): calcium phosphate 500, sodium chloride 74.0, potassium citrate 220, potassium sulfate 52.0, magnesium oxide 24.0, magnesium carbonate 3.50, ferric citrate 6.00, zinc carbonate 1.60, cupric carbonate 0.300, potassium iodate 0.0100, sodium selenite 0.0100, chromium potassium sulfate 0.550, sucrose 118.

^†^Vitamin mixture (AIN-76) (mg/kg): thiamin 600, riboflavin 600, pyridoxin 700, niacin 300, calcium pantothenate 160, folic acid 200, biotin 200, cyanocobalamin 10.0, retinol 24,000, cholecalciferol 2.50, tocotrienols 5,000, menadione 5.00, sucrose 979,000.
